# Biocontrol Using *Bacillus amyloliquefaciens* PP19 Against Litchi Downy Blight Caused by *Peronophythora litchii*

**DOI:** 10.3389/fmicb.2020.619423

**Published:** 2021-01-12

**Authors:** Li Zheng, Shilian Huang, Tom Hsiang, Guohui Yu, Dongliang Guo, Zide Jiang, Jianguang Li

**Affiliations:** ^1^Innovative Institute for Plant Health, College of Agriculture and Biology, Zhongkai University of Agriculture and Engineering, Guangzhou, China; ^2^Key Laboratory of South Subtropical Fruit Biology and Genetic Resource Utilization (MOA), Guangdong Province Key Laboratary of Tropical and Subtropical Fruit Tree Research, Institute of Fruit Tree Research, Guangdong Academy of Agricultural Sciences, Guangzhou, China; ^3^School of Environmental Sciences, University of Guelph, Guelph, ON, Canada; ^4^Department of Plant Pathology, Guangdong Province Key Laboratory of Microbial Signals and Disease Control, South China Agricultural University, Guangzhou, China; ^5^Chinese Academy of Tropical Agricultural Sciences Guangzhou Experimental Station, Guangzhou, China

**Keywords:** *Bacillus amyloliquefaciens* PP19, litchi downy blight, genome sequencing, comparative genomic analysis, preharvest treatment, biocontrol efficacy

## Abstract

*Bacillus amyloliquefaciens* has been widely used in the agriculture, food, and medicine industries. Isolate PP19 was obtained from the litchi fruit carposphere and showed biocontrol efficacy against litchi downy blight (LDB) whether applied preharvest or postharvest. To further understand the underlying regulatory mechanisms, the genome of PP19 was sequenced and analyzed. The genome comprised a 3,847,565 bp circular chromosome containing 3990 protein-coding genes and 121 RNA genes. It has the smallest genome among 36 sequenced strains of *B. amyloliquefaciens* except for RD7-7. In whole genome phylogenetic analysis, PP19 was clustered into a group with known industrial applications, indicating that it may also produce high-yield metabolites that have yet to be identified. A large chromosome structural variation and large numbers of single nucleotide polymorphisms (SNPs) between PP19 (industrial strain) and UMAF6639 (plant-associated strain) were detected through comparative analysis, which may shed light on their functional differences. Preharvest treatment with PP19 enhanced resistance to LDB, by decreasing the plant H_2_O_2_ content and increasing the SOD activity. This is the first report of an industrial strain of *B. amyloliquefaciens* showing a plant-associated function and with major potential for the biocontrol of LDB.

## Introduction

Litchi (*Litchi chinensis*) belongs to Sapindaceae and is native to South China. Currently, it is mainly distributed in China, India, Thailand, Vietnam, South Africa, Australia, and other tropical and subtropical regions (Tindall, 1994). Litchi is known in China as the “King of fruit,” with its taste and its high nutritional value; the pulp is rich in nutrients such as sugars, organic acids, dietary fibers, proteins, amino acids, polyphenols, and minerals (Jiang et al., 2006). During postharvest storage of litchi, the fruit can easily turn brown and rot due to litchi anthracnose, sour rot, and downy blight. Among these diseases, litchi downy blight (LDB) caused by *Peronophythora litchii* is the most serious (Wang et al., 2013). LDB alone may destroy 20–30% of litchi fruit every year (Zheng et al., 2019).

At present, the disease is mainly controlled by chemical methods. Due to the shortcomings of chemical control, such as issues arising from fungicide residues, biological control has become a desirable alternative. Pterostilbene, a phenolic compound found in plants, was found to inhibit the germination of *Peronophythora litchii* sporangia and affect the growth of its mycelia by deforming and shrinking the mycelia and sporangia, and damaging the cell wall, plasma membrane and organelles (Xu et al., 2018). Similarly, isoliquiritin, which is one of the flavonoid compounds found in licorice (*Glycyrrhiza uralensis*), can also damage the cell membrane of *P. litchii* (Luo et al., 2016). Zeamines, produced by *Dickeya zeae*, can damage the endomembrane of *P. litchii*, and zeamine-treated postharvest litchi fruit showed lower levels of *P. litchii* infection (Liao et al., 2015). Volatile organic compounds (VOCs) from *Streptomyces fimicarius* BWL-H1 can damage the endomembrane system and cell wall of *P. litchii* (Xing et al., 2018). Postharvest treatment of litchi fruit with *Bacillus amyloliquefaciens* LY-1 resulted in a lower pericarp browning index and percentage of fruit decay and higher fruit quality (Wu et al., 2017).

*Bacillus amyloliquefaciens* is used for commercial production of *Bam*H I restriction enzyme (Wilson and Young, 1975), the antibiotic barnase (Hartley and Rogerson, 1972) and other antibiotics (Yu et al., 2002), alpha amylase (Tanaka and Hoshino, 2003), and proteinase subtilisin (Peng et al., 2004). It can successfully colonize the epidermis of fruit and vegetables, compete with pathogens for nutrition, secrete antibacterial substances to inhibit the growth of pathogens, and induce plant defense systems to resist pathogen invasion for successful biological control (Hong et al., 2013). Increasing research has shown that *B. amyloliquefaciens* could be used in the biological control of postharvest diseases. *B. amyloliquefaciens* NCPSJ7 reduced the disease incidence of grape gray mold caused by *Botrytis cinerea* (Zhou et al., 2020). Iturin A produced by *B. amyloliquefaciens* BUZ-14 showed strong *in vitro* and *in planta* antifungal activity against *B. cinerea*, *Monilinia fructicola*, *Monilinia laxa*, *Penicillium digitatum*, *Penicillium expansum* and *Penicillium italicum*, which are major pathogens of postharvest fruit (Calvo et al., 2019). Several other strains of *B. amyloliquefaciens* are known to decrease postharvest diseases, such as 9001 against apple ring rot (Li et al., 2013), BA3 against pear gray mold (Qu et al., 2016), BGP20 against vegetable soft rot (Zhao et al., 2013), BUZ-14 against postharvest diseases of orange, apple, grape, and stone fruit (Calvo et al., 2017), CPA-8 against postharvest diseases of sweet cherry fruit (Gotor-Vila et al., 2017; Vilanova et al., 2018), and DH-4 against citrus green mold (Chen et al., 2018).

The genomes of several *B. amyloliquefaciens* strains have been sequenced, which allows for comparative genomic analysis and association of particular genes with particular functional uses. Based on marker gene and whole-genome sequence comparisons, *B. amyloliquefaciens* has been subdivided into two groups, called “industrial” and “plant-associated” (Borriss et al., 2011; Magno-Pérez-Bryan et al., 2015). The industrial group is commonly used as industrial producers of primary metabolites, including enzymes, purine nucleosides, riboflavin, among others (Zakataeva et al., 2007; Sheremet et al., 2011; Meng et al., 2019), while the plant-associated group is considered plant-associated due to the ability to promote plant growth and exhibit biocontrol activity against pathogens (Fan et al., 2012; Molinatto et al., 2016; Xie et al., 2020).

*Bacillus amyloliquefaciens* PP19 was isolated from the carposphere of litchi fruit pericarp and showed antifungal activity against *Peronophythora litchii* SC18, and LDB biocontrol with either preharvest or postharvest treatments (unpublished data). The VOCs produced by PP19, especially the compounds benzothiazole (BTH) and α-farnesene (AF), can inhibit the growth of *P. litchii* and reduce the severity of LDB (Zheng et al., 2019). The purpose of this study was to further investigate PP19, by sequencing its genome and characterizing its genes to shed light on its functional abilities.

## Materials and Methods

### Bacterial Culture and DNA Extraction

*Bacillus amyloliquefaciens* PP19 cells were cultivated at 28°C in 500 ml flasks, containing 100 ml of LB media for 24 h, and subsequently harvested through centrifugation at 10,000 × *g* for 5 min at 4°C. Genomic DNA was extracted immediately following Hoffman and Winston (1987). The purity and integrity of DNA were assessed by agarose gel electrophoresis and then quantified by Qubit^TM^ Fluorometer (Invitrogen, CA, United States).

### Genome Sequencing, Assembly, and Annotation

Genome sequencing and assembly were performed at Novogene Bioinformatics Technology Co., Ltd. (Beijing, China), on the Nanopore PromethION. All reads were assessed by NanoPlot with the threshold value Q > 7, and then the clean data were assembled with Unicycler (Wick et al., 2017). The coding genes of PP19 were predicted using GeneMarkS (Version 4.17) (Besemer et al., 2001). Interspersed nuclear elements and tandem repeats (TRs) were identified by RepeatMasker (Version open-4.0.5) (Saha et al., 2008) and TRF (Tandem Repeats Finder, Version 4.07b) (Benson, 1999), respectively. Transfer RNA (tRNA), ribosomal RNA (rRNA), and small RNA (sRNA) genes were detected by tRNAscan-SE (Version 1.3.1) (Lowe and Eddy, 1997), rRNAmmer (Version 1.2) (Lagesen et al., 2007), and CMsearch (Version 1.1rc4) (Cui et al., 2016) with default parameters, respectively. The prediction of genomic islands (GIs) and prophages was done with IslandPath-DIOMB (Version 0.2) (Hsiao et al., 2003) and phiSpy (Version 2.3) (Zhou et al., 2011), respectively. The clustered regularly interspaced short palindromic repeat (CRISPR) sequences were predicted by the CRISPR digger (Ge et al., 2016). For functional annotation, the predicted protein sequences were compared against the Gene Ontology (GO) database (Ashburner et al., 2000), Kyoto Encyclopedia of Genes and Genomes (KEGG) (Kanehisa et al., 2006), Clusters of Orthologous Groups of Proteins (COG) database (Galperin et al., 2015), Non-Redundant Protein Database (NR) (Li et al., 2002), Transporter Classification Database (TCDB) (Saier et al., 2016), Swiss-Prot (Bairoch and Apweiler, 2000), and Carbohydrate-Active enZYmes Database (CAZy) (Cantarel et al., 2009).

### Phylogenetic Analyses and Comparative Genome Analysis

Complete amino acid gene sets of 36 *B. amyloliquefaciens* strains were downloaded from the NCBI database.^1^ The whole genomes were aligned, and a genome-based phylogenetic tree was constructed using CVTree3 (Zuo and Hao, 2015). MUMmerV3.22 (Kurtz et al., 2004), and used for identification of homologous regions and for collinearity analysis with default parameters. LASTZ (Large-Scale Genome Alignment Tool) (Harris, 2007) was used for detecting InDels (insertions-deletions) and SV (chromosomal structural variation).

### Evaluation of Biocontrol Efficacy of Preharvest Treatment of Litchi Fruit With PP19

The pathogen *P. litchii* SC18 and the biocontrol agent *B. amyloliquefaciens* PP19 were cultured following Zheng et al. (2019). During summer 2019 in Danzhou city, Hainan province, litchi fruit of cv. “Feizixiao” at approximately 60% ripening was sprayed with PP19 at 5 × 10^7^ CFU/mL or 1/10 strength LB broth on the litchi trees until runoff. After 7 days, the fruit was harvested, and brought back to the laboratory, 30 litchi fruits were placed in each plastic container (32 × 22 × 10 cm), in which the bottom was covered with filter paper moistened with 15 mL sterile water. After 24 h, fruits were sprayed with 30–60 mL of *P. litchii* SC18 at 5 × 10^4^ sporangia/mL per container, and maintained in a greenhouse at 25°C with a day/night cycle of 12/12 h. The disease index was calculated following Zheng et al. (2019).

### Enzyme Activity Detection in the Pericarp

Fruit of litchi cv. “Feizixiao” was sampled in 2019. Nine fruits per treatment were sampled at each time point (0, 48, 84, 96, 108, and 120 hpi). Subsequently, the pericarps were collected and immediately packed with tin foil, frozen in liquid nitrogen, and stored at −80°C until the assays. The content of H_2_O_2_ and the activities of catalase (CAT, EC 1.11.1.6), superoxide dismutase (SOD, EC 1.15.1.1), and peroxidase (POD, EC 1.11.1.7) were determined with corresponding detection kits (Nanjing Jiancheng Biological Engineering Institute, Nanjing, China).

## Results

### Genome Sequencing of *B. amyloliquefaciens* PP19

**FIGURE 1:**
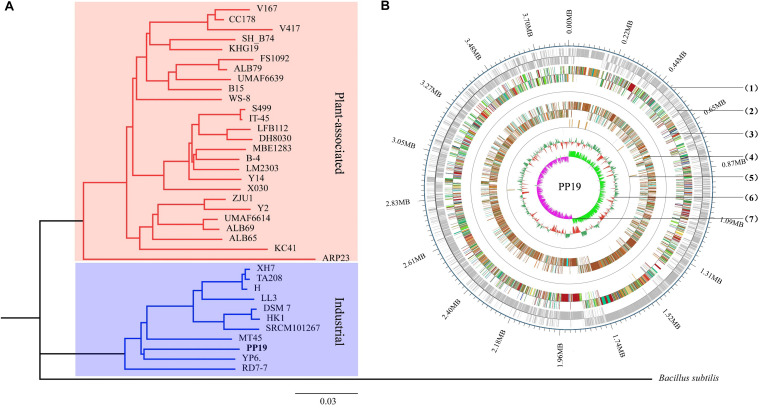
Phylogenetic analysis and circular chromosome of strain PP19. **(A)** Phylogenetic tree of PP19 based on the whole genome sequences of 37 *B. amyloliquefaciens* strains with *B*acillus *subtilis* used as an outgroup. **(B)** The circular genome map revealing the genetic basis of PP19. From outside to inside, the map shows the (1) position of the genome, (2) coding genes on the + and – strand, (3) COG annotation, (4) GO annotation, (5) non-coding RNA, and (6) GC content. The inner red indicates that the GC content in this region is lower than the genome-wide average GC content, while the outer green indicates the opposite. The higher the peak is, the greater the difference with the average GC content (7) GC skew value (G–C/G + C). When the value is positive, the CDS is more likely to be transcribed from the positive chain; otherwise, the CDS is more likely to be transcribed from the negative chain.

The Nanopore PromethION produced 342,009 reads with average read length of 13,054 bp. The 4.46 billion base pairs of reads, were assembled using Unicycler to generate a circular chromosome 3,847,565 bp in length with 46.27% GC content with no plasmids and 1160-fold genome coverage (Figure 1B and Supplementary Table 1). GeneMarkS was used to predict 3,990 protein coding sequences (CDSs), accounting for 89.85% of the genome, with an average length of 866 bp per gene (Supplementary Table 1). The results of genome component analyses showed that there were 226 interspersed repeats, 193 TRs, 86 tRNA genes, 27 rRNA genes, 8 sRNA genes, 8 GIs, and 6 prophages with no CRISPR sequences (Supplementary Table 2).

Functional annotation was based on diamond alignment of the protein sequences of predicted genes with several commonly used databases. In the COG analysis, 2,864 proteins were classified into 25 categories. Except for the prediction for the general function category (304 genes), the amino acid transport and metabolism (303 genes), and the transcription (279 genes) categories showed the highest percentages among 25 categories (Supplementary Figure 1).

GO analysis divided 2,684 proteins into three categories: biological processes, molecular functions, and cellular components. The metabolic process and cellular process, catalytic activity and binding, cell part and cell showed the highest gene abundance in the three categories, respectively (Supplementary Figure 2). In the KEGG pathway analysis, the metabolic pathway accounted for the highest proportion (Supplementary Figure 3). CAZy is a database of carbohydrate enzymes, including related enzyme families that catalyze the degradation, modification, and biosynthesis of carbohydrates. It contains five main categories: glycoside hydrolase (GH), glycosyl transferase (GT), polysaccharide lyase (PL), carbohydrate esterase (CE), and auxiliary activity (AA). There were 58, 52, 3, 15, 1, and 41 genes classified as GH, GT, PL, CE, AA, and CBM (carbohydrate-binding module) categories, respectively (Supplementary Figure 4). TCDB analysis indicated that the genes annotated as porters (169 genes, including uniporters, symporters, antiporters) and P-P-bond-hydrolysis-driven transporters (153 genes) accounted for 71.88% of all predicted transporters (448 genes) (Supplementary Figure 5).

### Phylogenetic and Comparative Genomic Analyses

Thirty-six *B. amyloliquefaciens* genome sequences were used in phylogenetic analysis. The characteristics of these genomes were enumerated (Supplementary Table 3), however, no obvious differences between plant-associated and industrially relevant strains were found in terms of genome size, number of genes, proteins, rRNAs, or tRNAs, while plant-associated strains showed a higher level of GC content (Supplementary Table 3). Aside from strain RD7-7, PP19 showed the smallest genome size, smaller by 3.85 to 4.24 Mb, but this did not result in a decreased number of predicted genes or proteins (Supplementary Tables 1, 3).

**FIGURE 2:**
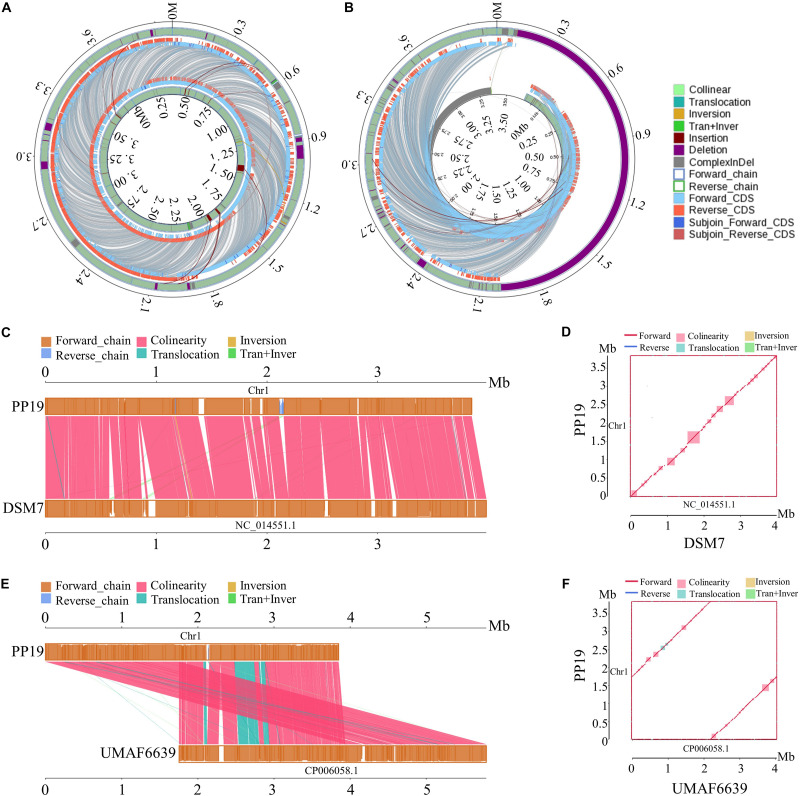
Chromosome structural variation (SV) and synteny analysis between PP19 and DSM7, PP19 and UMAF6619. **(A)** SV analysis between PP19 and DSM7, **(B)** SV analysis between PP19 and UMAF6619, **(C,E)** synteny analysis between PP19 and DSM7, PP19 and UMAF6619 (parallel collinear graph), **(D,F)** synteny analysis between PP19 and DSM7, PP19 and UMAF6619 (two dimensional collinear graph). Collinear, collinear region; Translocation, translocation region; Inversion, inversion region; Tran + Inver, translocation and inversion region; Insertion, region where the inserted fragment is greater than or equal to 50 bp; Deletion, region where the deleted fragment is greater than or equal to 50 bp; ComplexInDel, region that is unmatched but located in the same region; Forward_chain, the forward chain of the genome sequence; Reverse_chain, the reverse chain of the genome sequence; Forward_CDS, CDS translating on the forward chain of genome sequence; Reverse_CDS, CDS translating on the reverse chain of genome sequence; Subjoin_Forward_CDS, supplementary CDS translating on the forward chain of genome sequence; Subjoin_Reverse_CDS, supplementary CDS translating on the reverse chain of genome sequence; when the matching sequence of the deleted gene or inserted gene reaches 100% consistency, the matching sequence is considered a supplementary CDS.

The phylogenetic tree showed that PP19 clustered with the economically important strains named here as the Industrial Group (Figure 1A). However, PP19 was confirmed to have a biocontrol effect against LDB (unpublished data). To the best of our knowledge, this is the first report that a strain grouping with economically important industrial-use strains could be used in biocontrol of a plant disease. Comparative genomic analysis was applied to reveal the differences in the genome between PP19 and DSM7 (industrial strain characterized by its enormous potential to produce extracellular enzymes of industrials importance including amylases and proteases) and between PP19 and UMAF6639 (plant-associated strain). There were 174 indels detected between PP19 and DSM7, while 133 indels between PP19 and UMAF6639. A large number of SNPs was detected between PP19 and UMAF6639 (195,510), while only 43,045 were found between PP19 and DSM7 (Supplementary Table 4). Several long fragment deletions and insertions were detected between PP19 and DSM7, resulting in minor chromosome SV between them (Figure 2A). Chromosome SV analysis between PP19 and UMAF6639 indicated that almost half of their genomes were not matched (Figure 2B). Synteny and collinearity were examined between PP19 and DSM7 and between PP19 and UMAF6639. The results demonstrated that nearly all regions of sequence similarity fell along the diagonals of the forward strand, except for several gene sites, indicating a generally similar gene and sequence order between PP19 and DSM7 (Figures 2C,D). In a comparison of PP19 and UMAF6639 genomic sequences, several translocation sites were detected (Figures 2E,F).

### Preharvest Treatment With PP19 Suppressed LDB and Enhanced the Activity of Defense-Related Enzymes

Genome analysis further revealed the major traits of strain PP19 involved in the biocontrol activity against LDB. There were 12 gene clusters related to secondary metabolite biosynthesis in PP19 (Supplementary Table 5), including one lantipeptide, two non-ribosomal peptides (NRPS), one type III polyketide synthetase (PKS) and one other type of PKS, two terpenes, one TransAT-PKS, three TransAT-PKS-NRPS, and one other cluster (Supplementary Table 5). Therefore, it can be inferred that PP19 could suppress the growth of *P. litchii* by producing diverse secondary metabolites, and we observed *in vitro* antagonism (not published).

**FIGURE 3:**
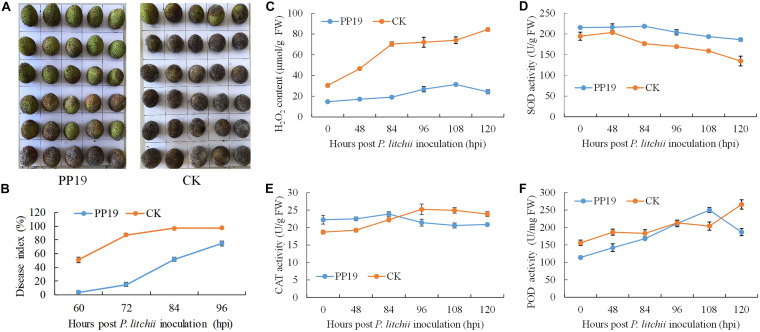
Suppression of LDB and enhancement of defense-related enzyme activity by preharvest treatment with PP19. **(A)** Visual comparison between PP19 preharvest treatment and control groups at 72 hpi. **(B)** LDB disease index for fruit treated with PP19 solution at preharvest and those treated with the control LB media. **(C–F)** Comparison of the H_2_O_2_ contents and SOD, CAT and POD activities detected after *P. litchii* inoculation in the PP19 preharvest treatment and control groups.

To validate its use in controlling LDB at the preharvest stage and the possible underlying mechanism, PP19 was tested on preharvest litchi fruit with clean LB solution as a control. After 7 days, the treated fruit was harvested and inoculated with spores of *P. litchii* SC18. The disease severity was recorded from 60 to 96 hours post-inoculation (hpi) at 12 h intervals, and the results showed a significantly lower disease index at 60, 72, 84, 96 hpi in fruit pretreated with PP19; however, the difference decreased with increasing time (Figures 3A,B).

To explore the effects of PP19 on plant resistance to LDB, the plant H_2_O_2_ contents and four related enzyme activities were assessed from the litchi pericarp. The H_2_O_2_ content in the litchi pericarp treated with PP19 was lower than that of the control after *P. litchii* inoculation. With increasing time, the H_2_O_2_ content of PP19-treated litchi pericarps showed a small increase, peaking at 108 hpi with a 2.1-fold increase, and then decreasing at 120 hpi. For the control, accelerated accumulation of H_2_O_2_ was detected before 84 hpi, followed by a slow increase (Figure 3C). The activity of SOD trended to decrease with increasing time in both the control and PP19-treated litchi pericarps, and the SOD activity in the PP19 treatment was higher than that in the control (Figure 3D). There was a slight increase in CAT activity before 84 and 96 hpi in the PP19 treatment and control groups, respectively, and the CAT activity decreased after peaking. The CAT activity in the PP19 treatment was higher than that in the control before 84 hpi but lower after 96 hpi (Figure 3E). The POD activity increased with increasing time in both the PP19 treatment and control groups, except at 120 hpi in the PP19 treatment, and the POD activity in the PP19 treatment was lower than that in the control, except at 108 hpi (Figure 3F).

## Discussion

The industrial group of *B. amyloliquefaciens* is often used industrially, as these strains are usually isolated from particular environments or generated by traditional mutation selection. Strains XH7 and TA208 were obtained by conventional mutagenesis, and these showed high yields of purine nucleosides, guanosine and ribavirin (Yang et al., 2011; Zhang et al., 2011). Strain LL3 was isolated from fermented food (Korean bibimbap) and could synthesize medicinally used poly-γ-glutamic acid (Geng et al., 2011), while RD7-7 isolated from rice doenjang (Korean fermented soybean paste) showed antimicrobial activity against *Bacillus cereus* (Eom and Choi, 2016). Strain HK1 was isolated from used agricultural mulch film, and exhibited a greater biodegradation of agricultural plastic film (Zhang et al., 2018). Strain YP6 was isolated from the rhizosphere of *Lolium perenne* from a phosphorus mine, and the alkaline phosphatase produced by YP6 showed high potential for biodegradation of organophosphorus pesticides (Meng et al., 2019). In this study, we sequenced the whole genome of *B. amyloliquefaciens* strain PP19 which was isolated from the carposphere of litchi fruit pericarp. Surprisingly, PP19 showed biocontrol activity against LDB but its genome was more similar to industrially relevant strains rather than plant-associated strains based on whole genome sequence comparisons.

We speculated that there were differences in genome structure and gene function between plant-associated and industrially relevant strains. Plant-associated strains showed a higher GC content, while no significant differences in genome size and gene number were observed between them (Supplementary Table 3). Comparative genomic analysis confirmed major chromosome SV between PP19 (industrial strain) and UMAF6639 (plant-associated strain) (Figure 2), which may result in functional differences. Comparative genome analyses of 37 genomes of *B. amyloliquefaciens* revealed differences which might be reflected in gene function. Unique genes common to the plant-associated clades are mainly found into the following COG categories: metabolism of amino acid transport and metabolism (E), carbohydrate transport and metabolism (G), synthesis of secondary metabolites (Q), and general function prediction (R) (Magno-Pérez-Bryan et al., 2015). COG analysis of the PP19 genome showed a similar classification (Supplementary Figure 1), which may indicate functional similarity between this and other plant-associated strains and partly explain the plant-associated function.

A previous study indicated that postharvest treatment with PP19 showed significant biocontrol against fruit LDB (unpublished data), and pre-exposure of postharvest litchi fruit to the VOCs produced by PP19 significantly reduced the severity of LDB (Zheng et al., 2019). Here, we assessed the biocontrol efficacy of preharvest treatment with PP19 to further evaluate its field application prospects, and the result confirmed PP19 showed a great potential for postharvest preservation (Figures 3A,B). H_2_O_2_, a representative reactive oxygen species that can be overproduced when induced by various abiotic and biotic stresses, is highly reactive and can cause damage to plants (Mhamdi and Van Breusegem, 2018). SOD, CAT, and POD are important enzymatic antioxidants for scavenging of reactive oxygen species (Gill and Tuteja, 2010). PP19 pretreatment with litchi resulted in a greater ability to scavenge H_2_O_2_ (Figure 3C), mainly due to the higher activity of SOD instead of CAT and POD (Figures 3D–F), as the SOD always showed a higher activity than control. The activity of CAT showed a little fluctuation in both control and PP19 treatment, so it may play a little part in LDB resistance. The activity of POD increased substantially in both control and PP19 treatment, which may play an important role in LDB resistance while not in difference between control and PP19 treatment.

Industrial strains tend to produce higher levels of a metabolite: XH7 (purine nucleoside guanosine) (Yang et al., 2011), TA208 (guanosine and ribavirin) (Zhang et al., 2011), LL3 (poly-γ-glutamic acid) (Geng et al., 2011), and MT45 (surfactin) (Zhi et al., 2017). In addition, a large number of genes were classified into amino acid transport and metabolism (E), coenzyme transport and metabolism (H) and secondary metabolite biosynthesis, and transport and catabolism (Q) categories. We speculate that PP19 should also produce a high yield of certain metabolites contributing to its antagonism of *P. litchii*, which can enhance the antioxidant related enzymes activities, and that will be a focus of future research.

## Conclusion

In conclusion, preharvest treatment with PP19 suppressed LDB, showing great potential for the exploitation and utilization of this strain. We sequenced the genome of *B. amyloliquefaciens* PP19, and whole genome phylogenetic analysis placed it with industrially relevant strains. This is the first report of a industrially relevant strain being used for plant disease control. Substantial chromosomal SV between PP19 and UMAF6639 was detected through comparative analysis, which may be one of the reasons for the functional differences between plant-associated and industrial strains. COG analysis indicated functional similarity between PP19 and plant-associated strains. From the above, it is speculated that a certain high-yield metabolite with inhibitory action against *Peronophythora litchii* was produced by PP19, and this metabolite should be further identified and studied.

## Data Availability Statement

The accession number of *Bacillus amyloliquefaciens* PP19 genome sequence is CP062984. All other datasets for this study are included in the article/Supplementary Material.

## Author Contributions

LZ, SH, GY, and DG conducted the experiments. LZ and SH analyzed the data and wrote the manuscript. TH revised the manuscript. ZJ and JL designed the experiments and revised the manuscript. All authors contributed to the article and approved the submitted version.

## Conflict of Interest

The authors declare that the research was conducted in the absence of any commercial or financial relationships that could be construed as a potential conflict of interest.

## Footnotes

1 https://www.ncbi.nlm.nih.gov/genome/
