# Supplementary material for: Biocontrol Using Bacillus amyloliquefaciens PP19 Against Litchi Downy Blight Caused by Peronophythora litchii
Source: Front Microbiol. 2021 Jan 12;11:619423. doi: 10.3389/fmicb.2020.619423 (PMC7835641; doi:10.3389/fmicb.2020.619423)
Supplement: Supplementary file 1 [file Data_Sheet_1.docx]

Supplementary Material

| **Supplementary Table S1.** Genomic features of strain PP19 | |
| --- | --- |
|  |  |
| Features | Value |
| Number of Reads | 342009 |
| Number of Bases (bp) | 4464795864 |
| Mean Read Length (bp) | 13054.6 |
| N50 Read Length (bp) | 15537 |
| Mean Read quality | 10.3 |
| Genome size (bp) | 3847565 |
| G+C content (%) | 46.27 |
| Gene Number | 3990 |
| Gene total length (bp) | 3456897 |
| Gene average length (bp) | 866 |
| GC content in gene region (%) | 47 |
| Gene/Genome (%) | 89.85 |
| Intergenetic region length (bp) | 390668 |
| GC content in intergenetic region (%) | 39.81 |
| Intergenetic length/Genome (%) | 10.15 |

| **Supplementary Table S2.** Genome component analyses of strain PP19 | | | | |
| --- | --- | --- | --- | --- |
| Non-coding RNA (ncRNA) | |  |  |  |
| Type | Number | Average_Length | Total_Length | In Genome(%) |
| tRNA | 86 | 77 | 6642 | 0.1726 |
| 5s rRNA | 9 | 115 | 1035 | 1.0716 |
| 16s rRNA | 9 | 1538 | 13842 |  |
| 23s rRNA | 9 | 2928 | 26352 |  |
| sRNA | 8 | 80 | 641 | 0.0167 |
| Genomics Islands (GIs) | |  |  |  |
| GIs_ID | Start | End | Length | GC% |
| GIs001 | 117270 | 152762 | 35493 | 44 |
| GIs002 | 476595 | 507864 | 31270 | 37.78 |
| GIs003 | 650013 | 661978 | 11966 | 37.36 |
| GIs004 | 1565999 | 1576061 | 10063 | 46.81 |
| GIs005 | 1776909 | 1801915 | 25007 | 39.39 |
| GIs006 | 2094348 | 2098902 | 4555 | 44.04 |
| GIs007 | 2131971 | 2149736 | 17766 | 40.13 |
| GIs008 | 3386982 | 3393823 | 6842 | 40.47 |
| Prophage |  |  |  |  |
| Prophage_ID | Start | End | Length | GC% |
| Prophage_1 | 35391 | 45903 | 10513 | 44.84 |
| Prophage_2 | 1186016 | 1213018 | 27003 | 46.68 |
| Prophage_3 | 1773796 | 1835773 | 61978 | 41.64 |
| Prophage_4 | 2097431 | 2165328 | 67898 | 42.35 |
| Prophage_5 | 2235985 | 2281596 | 45612 | 46.32 |
| Prophage_6 | 3035968 | 3055276 | 19309 | 47.04 |
| Interspersed Repeat |  |  |  |  |
| Type | Number | Total Length(bp) | Average length(bp) | In Genome(%) |
| LTR | 137 | 11649 | 85 | 0.3028 |
| DNA | 36 | 2100 | 58 | 0.0546 |
| LINE | 40 | 2639 | 66 | 0.0686 |
| SINE | 12 | 934 | 86 | 0.0243 |
| RC | 1 | 39 | 39 | 0.001 |
| Total | 226 | 17202 | 77 | 0.4471 |
| Tandem Repeat |  |  |  |  |
| Type | Number | Repeat Size(bp) | Total Length(bp) | In Genome(%) |
| Tandem Repeat (TR) | 193 | 6~459 | 16420 | 0.4268 |
| Minisatellite DNA | 162 | 10~60 | 13315 | 0.3461 |
| Microsatellite DNA | 1 | 6~6 | 45 | 0.0012 |

| **Supplementary** **Table S3**. Characteristic of *Bacillus amylolique*faciens genomes used for phylogenetic and comparative genomic analysis | | | | | | | | |
| --- | --- | --- | --- | --- | --- | --- | --- | --- |
|  |  |  |  |  |  |  |  |  |
| Type | Strain | Accession | Size (Mb) | GC% | Gene | Protein | rRNA | tRNA |
| Plant-associated | ALB65 | CP029069.1 | 4.04 | 46.4 | 3,960 | 3,785 | 27 | 86 |
|  | ALB69 | CP029070.1 | 4.05 | 46.5 | 3,956 | 3,783 | 27 | 86 |
|  | ALB79 | CP029071.1 | 3.98 | 46.4 | 3,855 | 3,678 | 27 | 86 |
|  | ARP23 | CP035899.1 | 4.02 | 46.5 | 4,079 | 3,109 | 27 | 86 |
|  | B15 | CP014783.1 | 4.01 | 46.5 | 3,927 | 3,734 | 27 | 88 |
|  | CC178 | CP006845.1 | 3.92 | 46.5 | 3,845 | 3,673 | 27 | 86 |
|  | DH8030 | CP041770.1 | 3.99 | 46.5 | 3,986 | 3,766 | 27 | 85 |
|  | FS1092 | CP038028.1 | 4.24 | 45.9 | 4,238 | 4,049 | 28 | 86 |
|  | IT-45 | CP004065.1 | 3.93 | 46.6 | 3,893 | 3,704 | 30 | 95 |
|  | KC41 | CP044444.1 | 4.12 | 46 | 4,168 | 3,844 | 27 | 86 |
|  | KHG19 | CP007242.1 | 3.95 | 46.6 | 3,868 | 3,689 | 28 | 88 |
|  | LFB112 | CP006952.1 | 3.94 | 46.7 | 3,868 | 3,675 | 31 | 94 |
|  | LM2303 | CP018152.1 | 3.99 | 46.7 | 3,953 | 3,765 | 27 | 86 |
|  | MBE1283 | CP013727.1 | 3.97 | 46.5 | 3,909 | 3,703 | 27 | 86 |
|  | S499 | CP014700.1 | 3.93 | 46.6 | 3,876 | 3,701 | 24 | 81 |
|  | SH-B74 | CP030097.1 | 4.04 | 46.5 | 3,953 | 3,751 | 27 | 86 |
|  | UMAF6614 | CP006960.1 | 4.01 | 46.5 | 3,900 | 3,729 | 27 | 82 |
|  | UMAF6639 | CP006058.1 | 4.03 | 46.3 | 3,918 | 3,731 | 27 | 82 |
|  | V167 | CP044360.1 | 3.9 | 46.4 | 3,904 | 3,615 | 18 | 74 |
|  | V417 | CP044359.1 | 3.91 | 46.5 | 3,919 | 3,480 | 24 | 83 |
|  | WS-8 | CP018200.1 | 3.93 | 46.5 | 3,854 | 3,653 | 27 | 86 |
|  | X030 | CP040672.1 | 3.95 | 46.6 | 3,876 | 3,690 | 27 | 84 |
|  | Y14 | CP017953.1 | 3.96 | 46.4 | 3,919 | 3,729 | 27 | 86 |
|  | Y2 | CP003332.1 | 4.24 | 45.9 | 4,271 | 4,035 | 29 | 86 |
|  | ZJU1 | CP041691.1 | 4.06 | 46.4 | 4,041 | 3,838 | 27 | 86 |
| Industrial applicability | DSM 7 | FN597644.1 | 3.98 | 46.1 | 4,076 | 3,826 | 30 | 93 |
|  | H | CP041693.1 | 3.95 | 45.9 | 4,111 | 3,915 | 27 | 86 |
|  | HK1 | CP018902.1 | 4 | 46 | 4,140 | 3,903 | 27 | 87 |
|  | LL3 | CP002634.1 | 4 | 45.7 | 4,085 | 3,902 | 22 | 72 |
|  | MT45 | CP011252.1 | 3.9 | 46.1 | 3,894 | 3,704 | 24 | 81 |
|  | RD7-7 | CP016913.1 | 3.69 | 46.3 | 3,691 | 3,497 | 27 | 86 |
|  | SRCM101267 | CP021505.1 | 4.07 | 45.9 | 4,175 | 3,964 | 27 | 87 |
|  | TA208 | CP002627.1 | 3.94 | 45.8 | 4,018 | 3,858 | 18 | 70 |
|  | XH7 | CP002927.1 | 3.94 | 45.8 | 4,029 | 3,857 | 21 | 75 |
|  | YP6 | CP032146.1 | 4.01 | 45.9 | 4,087 | 3,844 | 27 | 86 |

| **Supplementary** **Table S4.** Indel and SNP analysis of PP19 compared to DSM7 and UMAF6639 | | | |
| --- | --- | --- | --- |
|  |  |  |  |
| Type | Reference Name | DSM7 | UMAF6639 |
|  | Sample_name | PP19 | PP19 |
| Insertion and deletion (Indel) | Start codon insertion | 11 | 10 |
|  | CDS inside insertion | 76 | 52 |
|  | Stop codon insertion | 2 | 7 |
|  | Start codon deletion | 10 | 5 |
|  | CDS inside deletion | 70 | 53 |
|  | Stop codon deletion | 5 | 6 |
| Single nucleotide polymorphism (SNP) | Start_synonymous | 1 | 5 |
|  | Stop_synonymous | 34 | 167 |
|  | Start_nonsynonymous | 17 | 93 |
|  | Stop_nonsynonymous | 19 | 64 |
|  | Premature_stop | 40 | 231 |
|  | Synonymous | 29660 | 133478 |
|  | Nonsynonymous | 9153 | 42572 |
|  | Total_CDS_SNP | 38909 | 176500 |
|  | Total_Intergenic_SNP | 4136 | 19010 |
|  | Total_SNP | 43045 | 195510 |

| **Supplementary** **Table S5.** Secondary metabolism gene clusters in  *B. amyloliquefaciens* PP19 | | |
| --- | --- | --- |
|  |  |  |
| Cluster | Cluster_name | Gene_number |
| Lantipeptide | Cluster 2 | 21 |
| Non-ribosomal peptide synthetases (NRPS) | Cluster 1,11 | 95 |
| Polyketides synthetase (PKS, type III ) | Cluster 9 | 49 |
| Terpene | Cluster 4,8 | 52 |
| TransAT-PKS | Cluster 5 | 46 |
| TransAT-PKS-NRPS | Cluster 6,7,10 | 173 |
| OtherKS (other type of PKS) | Cluster 3 | 48 |
| Other | Cluster 12 | 46 |

**Supplementary** **Figure S1.** COG function classifications. A: RNA processing and modification; B: Chromatin structure and dynamics; C: Energy production and conversion; D: Cell cycle control, cell division, chromosome partitioning; E: Amino acid transport and metabolism; F: Nucleotide transport and metabolism; G: Carbohydrate transport and metabolism; H: Coenzyme transport and metabolism; I: Lipid transport and metabolism; J: Translation, ribosomal structure and biogenesis; K: Transcription; L: Replication, recombination and repair; M: Cell wall/membrane/envelope biogenesis; N: Cell motility; O: Posttranslational modification, protein turnover, chaperones; P: Inorganic ion transport and metabolism; Q: Secondary metabolites biosynthesis, transport and catabolism; R: General function prediction only; S: Function unknown; T: Signal transduction mechanisms; U: Intracellular trafficking, secretion, and vesicular transport; V: Defense mechanisms; W: Extracellular structures; X: Mobilome, prophages, transposons; Z: Cytoskeleton.

**Supplementary** **Figure S2.** Gene Ontology (GO) analysis. Biological process (from left to right): biological adhesion, biological regulation, cell proliferation, cellular component organization or biogenesis, cellular process, death, developmental process, localization, locomotion, metabolic process, multi-organism process, multicellular organismal process; negative regulation of biological process, hitrogen utilization, positive regulation of biological process, regulation of biological process, reproduction, reproductive process, response to stimulus; rhythmic process, signaling; viral reproduction; cellular component (from left to right): cell, cell junction, cell part, extracellular region, extracellular region part, macromolecular complex, membrane-enclosed lumen, organelle, organelle part, virion, virion part; molecular function (from left to right): Antioxidant activity, binding, catalytic activity, enzyme regulator activity, molecular transducer activity, nucleic acid binding transcription factor activity, protein binding transcription factor activity, structural molecule activity, transporter activity.

**Supplementary** **Figure S3.** KEGG pathway annotation. Cellular Processes (A): Transport and catabolism (1), Cellular community-prokaryotes (2), cell motility (3), cell growth and death (4); Environmental Information Processing (B): Signaling molecules and interaction (5), Signal transduction (6), Membrane transport (7); Genetic Information Processing (C): Translation (8), Transcription (9), Replication and repair (10), Folding, sorting and degradation (11); Human Diseases (D): Neurodegenerative diseases (12), Infectious diseases (13), Immune diseases (14), Endocrine and metabolic diseases (15), Drug resistance (16), Cardiovascular diseases (17), Cancers (18); Metabolism (E): Xenobiotics biodegradation and metabolism (19), Nucleotide metabolism (20), Metabolism of terpenoids and polyketides (21), Metabolism of other amino acids (22), Metabolism of cofactors and vitamins (23), Lipid metabolism (24), Glycan biosynthesis and metabolism (25), Energy metabolism (26), Carbohydrate metabolism (27), Biosynthesis of other secondary metabolites (28), Amino acid metabolism (29); Organismal Systems (F): Nervous system (30), Immune system (31), Excretory system (32), Environmental adaptation (33), Endocrine system (34), Digestive system (35), Aging (36).

**Supplementary** **Figure S4.** CAZy (Carbohydrate-Active enZYmes Database) annotation. AA: auxiliary activity; CBM: carbohydrate-binding module; CE: carbohydrate esterase; GH: glycoside hydrolase; GT: glycosyl transferase; PL: polysaccharide lyase.

**Supplementary** **Figure S5.** TCDB (Transporter Classification Database) annotation. 1.A: alpha-Type Channels; 1.C: Pore-Forming Toxins (Proteins and Peptides) ; 1.E: Holins; 2.A: Porters (uniporters, symporters, antiporters); 3.A: P-P-bond-hydrolysis-driven transporters; 3.B: Decarboxylation-driven transporters; 3.D: Oxidoreduction-driven transporters; 4.A: Phosphotransfer-driven Group Translocators; 5.A: Transmembrane 2-electron transfer carriers; 8.A: Auxiliary transport proteins; 9.A: Recognized transporters of unknown biochemical mechanism; 9.B: Putative transport proteins.
